# Adapting acute malnutrition treatment protocols in emergency contexts: a qualitative study of national decision-making

**DOI:** 10.1186/s13031-020-00293-x

**Published:** 2020-07-15

**Authors:** Naoko Kozuki, Mamoudou Seni, Amin Sirat, Omar Abdullahi, Mena Fundi Eso Adalbert, Marie Biotteau, Amelia Goldsmith, Sarah L. Dalglish

**Affiliations:** 1 International Rescue Committee, 1730 M St. NW, Washington, DC 20036 USA; 2 International Rescue Committee, Niamey, Niger; 3 International Rescue Committee, Maiduguri, Nigeria; 4 International Rescue Committee, Mogadishu, Somalia; 5 International Rescue Committee, Juba, South Sudan; 6 International Rescue Committee, Bamako, Mali; 7 Department of International Health, Johns Hopkins Bloomberg School of Public Health, Baltimore, USA

**Keywords:** Acute malnutrition, Nutrition, Food insecurity, Niger, Nigeria, Somalia, South Sudan

## Abstract

**Background:**

Each year, an estimated 17 million children suffer from severe acute malnutrition (SAM) and 33 million from moderate acute malnutrition (MAM), with many of the most severe cases found in extremely food insecure contexts or conflict situations. Current global outpatient treatment protocols for uncomplicated SAM and MAM, adapted by most countries for use at national level, call for SAM and MAM to be managed separately, however global-level stakeholders have recently begun evaluating simplified and/or combined protocols managing acute malnutrition.

**Methods:**

This study analyzes national policy discussions and decision-making around outpatient acute malnutrition treatment for uncomplicated cases in emergency situations in Niger, Nigeria, Somalia, and South Sudan. Data collection (March–July 2018) included semi-structured in-depth interviews with 50 respondents (*N* = 11–15 per country) from government, funding agencies, and implementing partners, as well as 11 global and regional stakeholders. We also conducted a document analysis (*N* = 10–15 per country and at global level) to situate debates and evaluate current policy. Data were analyzed iteratively using thematic content analysis.

**Results:**

We find that while combined/simplified protocols for outpatient management of uncomplicated cases of acute malnutrition are being used in emergency situations in all four countries, there is widespread confusion about protocol terminology and content, stemming from a lack of coherence at the global level. As a result, national-level stakeholders express diverse, if overlapping, rationales for modifying current protocols, which vary given the intensity and scope of the emergency. Without specific global-level guidance, combined/simplified protocols are often used on an ad hoc basis, although the processes for triggering them were at least nominally controlled at the national level. Decisions about when and where to enact “exceptional” modifications to country protocols were often based on inconsistent determinations of what constitutes an “emergency.” Respondents said more evidence is needed on both clinical and operational aspects of these protocols, and they awaited clear guidance from global norm-setting agencies.

**Conclusions:**

Based on these findings, global-level stakeholders should urgently improve coordination and communication around existing protocols. Standardized guidance based on the available evidence is required to clarify best practices for combined management of SAM and MAM, particularly in emergency contexts (which should be defined) and in situations of limited resources. Given the complexity of governance arrangements in conflict situations, both guidance and updates on research must be disseminated in a rational, systematic, and digestible way to the multiplicity of field actors.

## Introduction

Over 50 million children suffer from global acute malnutrition [1]. Conflict is a major driver of acute malnutrition, particularly in Africa, where countries experiencing protracted conflict have undernourishment rates twice as high as those not affected by conflict [2]. Acutely malnourished children have higher risks of infections, long-term developmental delays, and increased chronic disease risk [3, 4]. They are also at significantly increased risk of death; a study in rural Malawi observed that children with moderate acute malnutrition (MAM) had triple the risk of death compared to well-nourished children [5]. Undernutrition is the most common risk factor in child deaths and will continue to be a major public health concern as the world experiences conflicts and emergencies due to political crises, migration, climate change, and other causes [6].

**Fig. 1:**
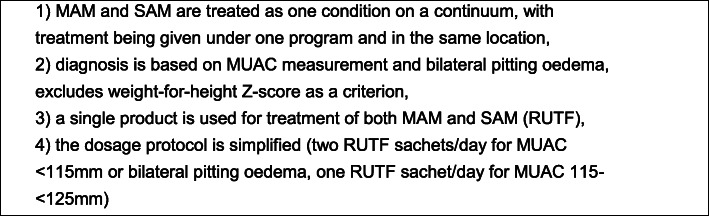
Major components of the Combined Protocol for outpatient treatment of uncomplicated MAM and SAM

Despite malnutrition being a condition that is on a continuum, from severe, to moderate, to not malnourished, global outpatient treatment protocols for uncomplicated acute malnutrition separate management of MAM and severe acute malnutrition (SAM), with the World Food Programme (WFP) taking responsibility for MAM treatment and UNICEF for SAM treatment. See Table 1 for a common case definition of uncomplicated MAM and SAM, although these definitions differ by context. These outpatient protocols are adapted by most countries for use at national level, including in emergency situations. Stakeholders have proposed a number of modifications to current approaches to address recognized operational difficulties, especially in conflict or crisis situations. For example, a “Combined Protocol” aims to integrate and streamline outpatient MAM and SAM treatment for uncomplicated cases by providing treatment for both at the same location, using simpler diagnostic criteria (see Fig. 1), a single ready-to-use product to treat both conditions, and a simplified dosage schedule [7, 8]. The Combined Protocol has been tested in a randomized controlled trial led by the International Rescue Committee (IRC) and Action Against Hunger (ACF), among other partners (results forthcoming). A number of other pilot projects and research studies are currently underway in Africa testing similar adaptations [9–12]. There are also earlier data, like those from a study conducted in 2013 in Sierra Leone of an integrated SAM/MAM treatment protocol using one product, showing non-inferiority of treatment outcomes and higher coverage compared to the existing program [9].

**Table 1:** Common case definition for severe and moderate acute malnutrition

Severe acute malnutrition	Moderate acute malnutrition
Mid-Upper Arm Circumference (MUAC) < 115 mm **and/or** Weight-for-height Z-score (WHZ) < − 3 **and/or** bilateral pitting oedema	MUAC ≥115 mm to < 125 mm **and/or** WHZ ≥ -3 to WHZ < − 2

Global-level technical documents have also proposed means of streamlining programming, optimizing cost-efficiency and supply management, and providing optimal care for children and families in emergency settings. While there is currently no guidance on MAM treatment from World Health Organization (WHO), a guidance note called the “MAM Decision Tool” circulated by the Global Nutrition Cluster used the term “expanded criteria” to refer to modifying (expanding) admissions criteria to admit MAM and SAM children into either an outpatient treatment programme (OTP) or targeted supplementary feeding programme (TSFP), when one or the other type of program is unavailable [13]. A Technical Issue Paper on a “Simplified Protocol” was also put forth by the Directorate-General for European Civil Protection and Humanitarian Aid Operations (ECHO), which similarly combines SAM and MAM treatment and simplifies diagnostic and treatment modalities [14].

Analyses of political, policy and decision-making processes around nutrition programming remain limited, despite it being understood that political factors – rather than environment or natural causes – are a key driver of hunger and malnutrition [15, 16]. Furthermore, countries experiencing conflict have unique governance challenges when it comes to decision-making and implementation of policies to combat undernutrition [17]. It is understood that policy choices in all health areas result from arguing and bargaining from amongst a set of choices, via negotiations between decentralized alliances or networks [18]. Yet little work has been done to consider how issue framing, ideas, and perceptions of current implementation can affect these decisions [19, 20]. Similarly, greater understanding is needed about how interactions between state actors and international agencies, including bilateral and multi-lateral agencies, aid and lending institutions, and non-governmental organizations (NGO) act as strong determinants of policy outcomes [21]. In the area of malnutrition, global and national actors do not always share the same frame for understanding the problem, causing difficulties in working together towards a coherent policy agenda [15].

We assess national decision-making around adaptations to outpatient acute malnutrition protocols for uncomplicated cases in emergency contexts. Among countries in which combined/simplified protocols have been introduced, discussed, and/or used in emergency settings, we selected Niger, northeast Nigeria (Borno State), Somalia, and South Sudan for this study. The sites vary markedly in terms of the scale of the malnutrition problem, the nature of the emergency, the strength and stability of government, and the resources available (Table 2). They are also at various stages in terms of the advancement of discussions on combined/simplified protocols. Yet in each of these countries, child malnutrition has reached emergency levels as a result of protracted civil wars (Somalia and South Sudan) or more localized insurgencies (Niger and Nigeria), crises that are often worsened by droughts and climate change. We examine the perspectives of in-country stakeholders regarding proposed modifications to current MAM and SAM treatment protocols.

**Table 2:** Descriptive overview of study settings

	Niger	Nigeria	Somalia	South Sudan
Population, in millions^1^	21.5	191	14.7	12.6
Gross national income per capita (current intn’l $)^1^	1000	5710	–	1440
Under-5 mortality rate^1^	91	104	133	91
Children < 5 stunted (%)^2^	42	44	25.3 (2009)	31 (2010)
Children < 5 wasted (%)^2^	10.3	10.8	15 (2009)	22.7 (2010)
Children < 5 underweight (%)^2^	31.7	31.5	23 (2009)	27.6 (2010)
Emergency context (at time of data collection)	Boko Haram insurgency in eastern Diffa state & unrest along on the Malian border are causing displacement and insecurity. Drought and poverty also habitually threaten food security.	Boko Haram insurgency in country’s northeast states since 2009. Over 2.5 M people internally displaced. High rates of MAM.	Civil war since 1991 concentrated in the south, which has killed half a million people. Groups including Al-Shabaab complicate this protracted conflict.	Civil war since 2013 following South Sudan’s independence. Fighting between government and opposition forces. Highly chaotic war causing prolonged food insecurity and extreme poverty.

Data sources: 1) World Bank Development Indicators, 2) WHO Global Health Observatory (GHO), 3) UNICEF Global Databases – Data are from 2016 to 2017 unless otherwise indicated

--: not available

## Methods

Case study methodology is used in policy analyses to understand and reconstruct phenomena holistically, and show links between context and underlying processes [22]. Case studies allow researchers to explore a single case’s internal logic and to compare and contrast phenomena across different countries, sites or areas [23]. We use case studies to understand decision-making around proposed adaptations to national malnutrition protocols with respect to the policy content, institutional environment, national context, and actor characteristics and relations [24].

Data collection (March – July 2018) followed standardized guidance in all four countries. Prior to in-country data collection, we performed a desk review of documents including national guidelines on management of acute malnutrition, other nutrition policies, strategic plans, implementation tools, and research reports. Information from documents was extracted to provide a synthesis of the four national policy environments, including the specific contents of existing national policies, any innovations being discussed or tested in country, and the institutional context around policies for treating acute malnutrition. Documents were also used to identify potential respondents for interviews.

Data collection in countries took place in capital cities (Niamey, Juba, Mogadishu, and Nairobi, where many Somalia satellite offices are based), except in Nigeria where it took place mainly in Maiduguri, Borno State (phone interviews were also conducted with Abuja-based respondents about the humanitarian response in the northeast). Semi-structured interviews were conducted by one data collector in Niger (*n* = 13 interviews, *n* = 15 interviewees) and Nigeria (*n* = 11 interviews and interviewees), and a second data collector in Somalia (*n* = 12 interviews and interviewees) and South Sudan (*n* = 10 interviews, *n* = 12 interviewees). Interview questions covered recent events in discussions on treatment protocols; the identities, roles, and positions of key actors; specific barriers or hesitations arising in discussions around modifying protocols; operational, financial, and/or practical considerations; and the importance of scientific evidence and/or global guidance in modifying national policies. Most interviews took place in person, though some took place by phone. Respondents included personnel of the Ministry of Health (MOH), UNICEF, WFP, and various NGOs in most countries. Interviews were also performed with select global and regional stakeholders regarding discussions at these levels as they impacted national-level policy decisions (*n* = 8 interviews, *n* = 11 interviewees). Interviews lasted an average of 54 min (range: 33–82 min) and were conducted in English, except for respondents from Niger and some global-level respondents, who were interviewed in French. Notes were taken for the small number of respondents who declined to be recorded; all other interviews were transcribed verbatim, then verified and completed by interviewers with notes on setting and non-verbal gestures.

Iterative data analysis began with regular debriefing discussions between data collectors to discuss emerging themes and adjust data collection as required, and combined analytical strands from interviews and the document review. A standardized coding form was used to systematically extract data from documents, allowing for comparison across cases of categories including policy content, contextual factors, stakeholder identities, and policy processes [25]. Following in-country data collection, de-briefing sessions were used to seek clarifications from IRC’s country nutrition focal points. Interview transcripts were analyzed using thematic coding, with NVivo software (version 11). The final codebook included both a priori categories based on the theoretical literature and research questions and in vivo codes emerging after testing the coding structure on a first set of interviews, and covered countries’ current malnutrition protocols; the origins, rationale, and arguments for or against combined/simplified protocols; national context; policy processes; and policy interactions at country, regional, and global level. Preliminary results were presented to in-country stakeholders in Niger and Somalia and a synthesis report was made available for review to all interviewees and in-country IRC staff in all four countries for accuracy of facts and interpretation prior to the drafting of this manuscript.

Ethical clearance was provided by the International Rescue Committee’s Institutional Review Board (IRB) (protocol #: H 1.00.016) and by the relevant bodies in all four countries.

## Results

In all four countries, discussions of combined/simplified protocols had taken place in key policy circles, notably the Nutrition Cluster (coordination mechanism for humanitarian response around nutrition), and were in limited use under exceptional circumstances. However policy trajectories varied considerably between Niger and Nigeria, where combined/simplified protocols were less accepted and used under more limited circumstances, and Somalia and South Sudan, where use of novel protocols was both more widespread and more codified. In Niger, discussions were propelled mainly by global actors, who faced reluctance from MOH on the issue of MUAC-only diagnosis. Stakeholders reported that discussions were nonetheless ongoing, and suggested that small-scale, under-the-radar uses of combined/simplified protocols were taking place in the conflict-affected Tillaberi and Diffa regions. In Nigeria, discussions were driven by UNICEF in response to the crisis in the northeastern states. An adapted protocol was tested in a small pilot in areas newly liberated from Boko Haram; NGOs had also implemented various components of novel protocols in Borno State. At federal level, these adaptations were being considered under guideline revisions process taking place at the time of this study.

In Somalia and South Sudan, novel protocols were more widely used, in part given the larger burden of food insecurity and logistical challenges in these countries. In Somalia, UNICEF rolled out what was usually referred to as the “Expanded Criteria” in five districts with high caseloads, in consultation with WFP, in late 2017. Use of the “Expanded Criteria” was accepted by malnutrition stakeholders, and used on an “exceptional” (if not infrequent) basis by implementing agencies. To use this protocol, agencies needed to obtain permission from the Nutrition Cluster, first from the Cluster lead by email, then after discussion in a Cluster meeting including MOH. In South Sudan, the “Expanded Criteria” were introduced by UNICEF and/or WFP (respondents’ recollections varied) through the Cluster meeting, using a slightly modified version of the protocol from the “MAM Decision Tool” [13]. As in Somalia, the Cluster granted approval to use the “Expanded Criteria” on an exceptional basis, however the process was more formalized; partners needed first get permission from the Cluster, then to specifically inform MOH, UNICEF, and WFP so that pipeline issues could be managed. Rapid Response Mechanisms also used the “Expanded Criteria.”

### Confusion about terminology and content of novel protocols

Respondents described adaptations to existing malnutrition protocols using varied terminology, and definitions were not necessarily consistent among actors and across countries. The most commonly used terms were “Combined Protocol” (used by partners involved in its randomized controlled trial), “Simplified Protocol” (used in some donor documentation) and “Expanded Criteria” (frequently used by WFP and codified in the “MAM Decision Tool”) (Table 3). However, respondents’ use of these terms often did not correspond to fixed definitions. When asked, respondents gave definitions focusing on one aspect of proposed policy modifications, such as the type of product used or diagnostic criteria. Some respondents used the terms interchangeably, and others with varying levels of precision. Many seemed confused:

“So I want to ask you, in terms of the extended admission criteria and … the combined protocol, is this the same or is it different? …. For me I understand it’s separate. So I want to have more information.” (International NGO, Nigeria).

“I wonder though whether it’s not just the same thing called different things.” (Bilateral agency, South Sudan).

**Table 3:** Indicative summary of novel protocols and/or guidance for management of acute malnutrition mentioned in this study

	Description	Relevant documents
Combined protocol	Protocol that treats SAM and MAM as one condition on a spectrum, with 1) treatment dispensed at a single location, 2) diagnosis on MUAC and oedema, 3) treatment with one ready-to-use food product, 4) simplified dosage.	Study protocol published in *Trials* (Bailey et al., 2018); ENN publication on Phase 1 (Bailey et al., 2016)
Simplified protocol	Protocol meant to increase flexibility for programming in exceptional emergency contexts that provides for 1) use of MUAC and oedema only for admission, follow-up and discharge; 2) use of a single ready-to-use food product; and 3) screening & provision of treatment provided at every contact with the community.	ECHO’s June 2017 technical Issue paper (ECHO, 2017) & Technical Annex; see FEX article in continuum of care issue
Expanded criteria	Protocol focusing on situations in which either an OTP, a TSFP, or both, are unavailable. Calls for a MUAC or WHZ-based dosing schedule of two sachets of the available product for SAM and one sachet of the available product for MAM per day.	Guidance note on CMAM in Emergencies; Global Nutrition Cluster MAM Task Force’s “Moderate Acute Malnutrition: a Decision Tool for Emergencies,” updated in 2017

All three terms (Combined Protocol, Simplified Protocol, and Expanded Criteria) were at least mentioned or recognized in all four countries.

Depending on the speaker, the term “Combined Protocol” was associated with 1) treating MAM (Niger, Nigeria), 2) using RUTF only to treat SAM and MAM children (Nigeria, Somalia), 3) using either product to treat both children (some respondents in all countries), and/or 4) using MUAC and oedema only to screen children (all countries). Respondents not familiar with the technical details appeared to infer from the name:

“The way I understand is that the Combined [Protocol] is the treatment of both moderate and severe acute malnutrition, where it will be combined together.” (International NGO, South Sudan).

The “Combined Protocol” was also sometimes explicitly associated with a prospective cohort study in Somalia and randomized controlled trial in Kenya and South Sudan being run by partners including the IRC. The “Expanded Criteria” was also defined in different ways, though often associated with changing screening cut-offs using MUAC. In Niger and Nigeria, this was understood as necessarily expanding treatment to include MAM children. A Nigerian federal health official provides a typical explanation using this definition:

“Expanded Criteria starts from the screening … for admission for the SAM program. It used to be below 11.5 … [expanding] it to some cases where you have MAM kids. Of course, between 11.5 and 12.5 is MAM.” (Government official, Nigeria).

However, in Somalia the “Expanded Criteria” seemed to refer to keeping SAM children in OTP treatment (in the absence of TSFP) until reaching MUAC discharge criteria of 12.5 cm, and theoretically the reverse (treating MAM children in OTP in the absence of TSFP), though this latter situation rarely seemed to occur in practice. In South Sudan, the “Expanded Criteria” was similarly defined as a “stopgap” provision for the same situation, summarized in a researcher’s notes from an unrecorded interview (Multi-lateral agency, South Sudan) as what is proposed in the “MAM Decision Tool”:

When there is OTP but no TSFP, 1 RUTF given for MAM kids.

When there is TSFP but no OTP, 2 RUSF given for SAM kids.

Respondents at global and regional level, and some respondents at country level, said the definitional confusion in countries stemmed from the lack of unified messaging at the global level. In the absence of WHO guidelines on combined or integrated management of acute malnutrition, there exist multiple proposed protocols, without strongly-defined guidance on when and where they should be implemented:

“The way that the Combined Protocol has been sold or presented and who’s presented it and how it’s been articulated has varied significantly from place to place.” (Bilateral agency, global level).

“I don’t think it’s being sensitized in all the countries in a systematic way.” (Bilateral agency, global level).

These definitional issues did not necessarily affect field-level decision-making about aspects of novel protocols, however the lack of shared understanding of terms may have complicated discussion between partners and at national level.

### Rationale of protocol adaptations differs by context

Across countries, a number of rationales were advanced for adopting combined/simplified protocols or specific aspects thereof. These included “saving lives” when the usual protocol could not be implemented, continuing service despite stock-outs, treating MAM to prevent SAM, using MUAC and oedema only for diagnosis to address limited human resource capacity, and improving efficiency and cost-effectiveness. The frequency with which these rationales were invoked varied by country, with a pronounced difference between Niger and Nigeria, versus Somalia and South Sudan (Table 4).

**Table 4:** Rationales as perceived by interviewees for using combined/simplified protocols for management of acute malnutrition

	Niger	Nigeria	Somalia	South Sudan
“Save lives” when the normal protocol is not possible	–	–	+++	++
Continue service in case of stock-outs	+	+	++	–
Treat MAM to prevent children from developing SAM	+++	+++	++	++
Not enough capacity for WHZ, so MUAC and oedema-only is necessary	–	–	+	++
Improve efficiency and/or cost-effectiveness of protocol	+	+	–	–

+++ Mentioned by many/most respondents as a rationale

++ Mentioned by some respondents as a rationale

+ Rarely mentioned as a rationale

- Not mentioned as a rationale

In all countries, respondents frequently pointed out that expanding the treatment criteria to allow for earlier inclusion, and/or treating MAM children (in Niger and Nigeria), had the benefit of reducing the SAM caseload. Many further noted that catching MAM before it became SAM both reduced the mortality danger for children and was less expensive and resource-intensive for providers. This rationale was invoked in all contexts, but most frequently in Nigeria and Niger, since these high-burden countries were not providing MAM treatment for all children at national level. One respondent in Niger observed:

“Once it’s stated that you’re not treating MAM, the number of SAM [cases] increases, so then, you have to start asking yourself questions. Are we not missing the mark?” (International NGO, Niger).

However, some respondents also feared that expanding treatment for MAM children would reduce available resources for SAM children, who were already not sufficiently treated, as some funding, attention, staff time, and other resources would be shifted away from them:

“I was like, ‘OK, if we are still below 60% of coverage [of SAM] in some cases, do you think this is the good decision to start doing the Expanded Criteria?’” (Multi-lateral agency, Nigeria).

Nonetheless, respondents in all countries recognized the ethical, clinical, and economic rationale for catching malnutrition cases earlier (i.e. in the MAM phase) to prevent children from developing SAM.

While this rationale of catching cases earlier was put forth in Somalia and South Sudan, stakeholders in these countries talked more about the impetus to “save lives” in difficult humanitarian conditions. As noted, these countries suffer from more protracted and widespread conflicts and greater food insecurity than Niger and Nigeria. Respondents frequently invoked some version of the phrase “save lives,” or used other morally-tinged language, when discussing reasons to adopt combined/simplified protocols:

“The intention is to save the children.” (Government official, Somalia).

“So you need to limit … some of the components and then take what you can for the sake of the children to receive the service.” (International NGO, South Sudan).

Other rationales, such as continuing service during stock-outs, using MUAC-only screening to deal with human resource constraints, and improving cost efficiency were mentioned to a somewhat lesser degree. The rationale of continuing service during stock-outs was most frequently discussed in Somalia, where respondents agreed that the Expanded Criteria could be used to cope with stock-outs occurring in an emergency situation (due to looting, supply delivery being hampered, etc.), however not to cover general stock-outs caused by poor logistical planning on the part of the implementing partners (even though this reportedly occurred with some regularity). The rationale of using MUAC-only screening due to human resource constraints was invoked somewhat less frequently, perhaps due to ongoing scientific disagreement about whether it is an adequate clinical measure to diagnose acute malnutrition.

Finally, a minority of respondents, frequently those who were more familiar with combined/simplified protocols (most often through exposure via networks in their NGOs or international organizations), said these protocols would increase efficiency and reduce health worker workload and overall expenditure. This rationale was more commonly mentioned in Niger and Nigeria than in Somalia and South Sudan. These respondents said these protocols reduced the number of SAM children, who require longer stays and more attention, by catching cases at the MAM level:

“If we manage this at the MAM level, that means there will also be more value for money because MAM treatment is completely cheaper than the treatment of SAM.” (Multi-lateral agency, Nigeria).

Rarely, the question of consumables (RUTF) and their cost was used as an argument in favor of considering novel protocols:

“I think it’s actually the supply constraints that should be pushing partners to think about alternative solutions.” (International NGO, Niger).

In this stakeholder’s view, difficulties funding adequate supplies of RUTF in Nigeria are seen as a reason to treat MAM, to reduce need for this product by reducing future SAM cases.

### Protocols are for use in emergencies, but what constitutes an emergency?

In all countries, combined/simplified protocols were associated with use in emergencies, and their use viewed as a temporary stop-gap measure not meant to supersede national guidelines. While government-led services follow standard national guidelines on acute malnutrition, combined/simplified protocols are used to the greatest extent by implementing partners in situations where the state has little to no oversight (i.e. in the most remote and conflict-affected settings, such as Al-Shabaab-controlled areas in Somalia). Where these protocols were used, they were frequently framed as an ad hoc, temporary solution used only while waiting for conditions to return to normal:

“… [M] aybe there is a fighting going on and we could not supply some emergency, it’s an extreme case … It’s a very short duration of two to three weeks, three to four weeks maybe.” (International NGO, South Sudan).

“For an emergency, you need a protocol to act in an emergency.” (Government official, Niger).

The implications of combined/simplified protocols in terms of increased need for product (usually RUTF) were seen by many respondents as an additional reason to limit implementation to emergency situations. In Somalia and South Sudan, respondents said combined/simplified protocols should be used on an exceptional basis because interchanging RUTF and RUSF made it difficult for UNICEF and WFP to forecast their supplies:

“It cannot be used for a long time, because it may – you know, WFP and UNICEF normally estimate their supplies from the beginning of the year.” (Multi-lateral agency, South Sudan).

While respondents in all countries agreed these protocols were appropriate for “emergency” situations, exactly what these consisted of was not always clear. While all could agree that outright conflict constituted an emergency, as did “insecure” conditions, definitions could nonetheless vary significantly by respondent, and by context. In the Tillaberi region of Niger, one stakeholder identified the three types of “insecurity” faced in that region:

“First there’s terrorism, along the Malian border where people attack visible manifestations of the State. There’s also banditry where health centers are attacked by armed bandits. The third one in our zone is animal attacks – hippopotamuses.” (International NGO, Niger).

Nor did criteria for defining an “emergency” necessarily overlap with stated criteria for triggering use of combined/simplified protocols (Table 5). The question of whether famines or seasonal peaks in malnutrition counted as an “emergency,” for example, was an open one, particularly in Niger, where high rates of acute malnutrition occur frequently outside of conflict areas. Given recurrent famines, one respondent said the current protocol was already designed specifically with food crises and/or nutrition emergencies in mind:

“The national protocol in itself is already an emergency document.” (International NGO, Niger).

**Table 5:** Criteria for using the combined protocol vs. emergency

	*According to a majority of informants:*	
	Defined as an “emergency”	Reason for using the combined protocol
Active conflict or insecure zone	Yes	Yes
Newly accessible zone with high rates of acute malnutrition	Yes	Yes
Stock-outs of RUTF and/or RUSF	No	?
Peak of malnutrition during the lean season	?	No
Areas where this is only an OTP or a TSFP (not both)	No	Yes

Whereas in Somalia, there was a general push to move away from the use of “emergency” language to better emphasize investments in sustainable and resilient systems for food and general insecurity. In South Sudan, despite the strict rules for implementing agencies to receive permission from the Nutrition Cluster before implementing the Expanded Criteria, what constitutes as “emergency” was not documented or codified anywhere.

Stakeholders generally restricted discussions about combined/simplified protocols to their use in emergency settings, despite the confusion around what constituted an “emergency.” Few respondents in any country made mention of adopting combined/simplified protocols in non-emergency situations.

### More evidence is needed before decisions can be made to modify protocols at national level

There was general agreement that while combined/simplified protocols presented inherent advantages, there was still a lack of or poor synthesis of existing evidence to formally support their integration into national protocols. As a whole, national policymakers and staff at NGOs and U.N. organizations frequently seemed convinced by the concept of combining and/or simplifying MAM and SAM protocols, and appeared to await evidence that would give them permission to do so:

“I think we need more scientific evidence … The Combined Protocol is better than separate protocols if there’s evidence.” (International NGO, Nigeria).

“If scientific-wise, it is very agreed that we can go for management of acute malnutrition using one product … For us, it would be very easier. Even for me, it will be easier for the management purpose and all the process of logistics and so on and so forth … if really it is well verified, cross-checked, and agreed.” (International NGO, South Sudan).

Respondents also called out aspects of these protocols which required further evidence, including both clinical aspects, having to do with safety and effectiveness, and operational aspects (Table 6). Regarding the latter, novel protocols’ cost implications were frequently evoked by respondents in all countries. As one respondent in Niger said,

“The problem is that in Niger, no one has calculated what you gain by using this protocol and what you lose by using this other protocol. As long as it hasn’t been calculated, it will be a bit difficult to convince people to use this protocol.” (Multi-lateral agency, Niger).

**Table 6:** Required research around combined/simplified malnutrition protocols

Type of research	Suggested topics
Clinical research	• Safety and effectiveness of using RUTF to treat MAM children and/or RUSF to treat SAM children (depending on the proposed protocol) • Appropriateness of using MUAC as a screening tool for “tall, slim” body types, and overlap with biochemical markers of malnutrition • (Minimum) dosage of RUTF/RUSF required for recovery • Impact of combined/simplified protocols on length of stay
Operational / implementation research	• Operational and costing implications of combined/simplified protocols • Impact of combined/simplified protocols on amount of product used • Pilot studies in every country to test specific protocols

Indeed, only studies of specific proposed combined/simplified protocols could provide these types of operational details, as recognized by this global-level respondent:

“That’s why you have to go with a pilot approach, because today governments have the same questions as management at global agencies. ‘Yes, but what will be the impact? Even if there’s a reduction over time how do we deal with the increase in the first months? What should we anticipate? How much will it cost?’ Etc, etc. And today, we’re a bit uncomfortable because we don’t have clear answers.” (Multi-lateral agency, regional level).

Pilot projects were underway on various combined/simplified protocols, or related issues, in all four countries, as respondents were well aware (Table 7). However, in some cases, it was difficult to discern whether so-called “pilot projects” were documenting their process and outcomes, or whether they were simply using alternative protocols or adaptations due to logistical constraints. This was particularly true when speaking of the areas most affected by conflict and insecurity in Niger and Nigeria, as well as in Somalia and South Sudan.

**Table 7:** Pilot projects and operational research mentioned by respondents in this study

Niger	Nigeria	Somalia	South Sudan
• “Mother MUAC” in Ouallam (ALIMA, IRC) • iCCM plus nutrition (ACF, World Vision) • Combined/ simplified protocol in Diffa (MSF)	• Using expanded criteria in 5 LGAs in Borno State (UNICEF) • Expanding admission criteria to 120 mm (MSF) • Small facility-based TSFP pilot (WFP)	• Expanded Criteria in 5 districts (UNICEF + WFP) • “Resiliency” project on putting TSFP, OTP in same location (UNICEF, WFP, MOH) • Mother MUAC • iCCM plus nutrition (ACF) • Combined Protocol (IRC)	• Pea-based RUSF (WFP) • Combined protocol (ACF, with support from IRC) • iCCM plus nutrition among low-literate CHWs (IRC)

Respondents were also aware to varying degrees of research currently under on aspects or versions of the combined/simplified protocols, particularly the OptiMA trials in Burkina Faso and elsewhere in West Africa (most frequently mentioned in Niger and Nigeria) and the trial of the “Combined Protocol” in Kenya and South Sudan run by IRC and ACF, alongside other partners. The results of these trials appeared to be eagerly awaited:

“So by and large, everyone is aware about the study and … everyone is waiting for the results.” (International NGO, South Sudan).

WFP has also recently produced impact evaluations of its programs on MAM treatment in humanitarian settings in the Sahel that will likely contribute to these discussions [26]. Finally, respondents mentioned Alliance for International Medical Action’s (ALIMA) work on MUAC and oedema-only diagnosis and MUAC screening by caregivers (“Mother MUAC”) [27], and referred to additional studies conducted by International Medical Corps, ACF and IRC.

## Discussion

Conflict is a large driver of hunger, malnutrition, and child deaths in many sub-Saharan countries. The innovations proposed by combined/simplified protocols to manage acute malnutrition in children in emergency settings were generally viewed positively by stakeholders working on humanitarian interventions in Niger, Nigeria, Somalia, and South Sudan, who said they made treatment more accessible for beneficiaries and logistically simpler to implement. While considerable confusion remained about the terminology and content of specific protocols, and the conditions for their use, national-level stakeholders appeared convinced by the various rationales put forward to support their use. In all countries, stakeholders were convinced treating MAM would help prevent children from developing SAM. In Somalia and South Sudan, respondents also frequently mentioned of these protocols’ potential to “save lives” by reaching more children; whereas in Niger and Nigeria, respondents more frequently mentioned their operational and logistical advantages. Nonetheless, particularly in Niger and Nigeria, respondents said they were awaiting further scientific evidence before they could advocate for formal adoption, specifically around issues of clinical effectiveness, operational implications, and cost.

Taken together, these four case studies reveal a lack of clear, coherent guidance from global level about when and whether to implement adapted protocols for management of acute malnutrition. Combined/simplified protocols were used in “exceptional” circumstances in all countries, and restricted to cases when the standard (national) protocol was unable to be implemented for some reason. In all countries, stakeholders from a multiplicity of organizations, including MOH (sometimes federal and state), UNICEF, WFP, and diverse international and national NGOs, worked together to attempt a coordinated response. Yet the processes for adopting combined/simplified protocols varied significantly by context, with a notable division between the more politically volatile and food-insecure countries, with more widespread and severe emergencies, and the more stable ones. In Somalia and especially South Sudan, the process for triggering use of a combined/simplified protocol was more formalized, requiring approval from the national Nutrition Cluster based on a specific set of criteria. In Niger and Nigeria, where conflicts were more localized and central governments better established, uses of combined/simplified protocols were used on a pilot or ad hoc basis. The complexity of the network of stakeholders engaged in the ultimate decision of triggering a combined/simplified protocol highlights the importance of greater national and global coordination, as well as clarity in messaging to ensure frontline health workers have appropriate guidance to execute their life-saving work in challenging contexts.

Work is currently underway at global level both to generate the scientific and programmatic evidence needed to strengthen current guidelines and recommendations, and to provide coherent, unified guidance from the various international agencies involved in responding to situations of acute malnutrition, including in emergency settings. Ongoing research on combined/simplified protocols includes randomized control trials such as ComPAS in Kenya and South Sudan (ISRCTN30393230) and OptiMA in the Democratic Republic of the Congo (NCT03751475), alongside a number of pilot and operational projects by U.N. agencies and NGOs such as Médecins Sans Frontières, ALIMA, and ACF, among others [10, 12, 28–31]. Actors at a number of U.N. agencies, global funding bodies, and NGOs have recently come together to discuss the emerging set of options for combining and/or simplifying the MAM and SAM protocols, particularly in the context of emergency settings. In March 2019, representatives of WHO, UNICEF, the United Nations High Commissioner for Refugees, and WFP met in Geneva to review and endorse simplified approaches for child wasting, calling for further evidence generation and a more harmonized approached to child wasting [32]. Such high-level efforts are a welcome response to the lack of a clear line on this issue perceived by field-level actors.

While combined/simplified protocols are generally seen as being designed for use in humanitarian emergencies, national stakeholders had divergent ideas about what constituted an emergency. Most or all stakeholders agreed that active conflict or newly liberated zones both counted as “emergencies” and were appropriate situations for using combined/simplified protocols. However, while respondents said that stock-outs of supplies such as RUTF and the lack of certain facilities (i.e. OTPs or TSFPs) could be reasons for triggering novel protocols, they also said these did not constitute an emergency. Conversely, when it came to peaks of malnutrition during the lean season, respondents tended to agree that this was not a reason to use combined/simplified protocols, but disagreed as to whether or not this constituted an “emergency.”

Definitional confusion about what constitutes an “emergency” is nothing new [33]. The U.N. Office for the Coordination of Humanitarian Affairs has defined an emergency as a “sudden and usually unforeseen event that calls for immediate measures to minimize its adverse consequences,” and a complex humanitarian emergency as “a humanitarian crisis in a country or region where there is considerable or total breakdown of authority result from internal and/or external conflict and which requires an international response that goes beyond the mandate or capacity of any single agency.” [34, 35] Crude mortality rates (e.g. > 1/10,000 persons/day) are also often also used to determine the need for a humanitarian response [33]. However, there is no agreed-upon definition of “emergency,” a fact related to the division and lack of coordination between “emergency” actors and other actors in health and development more broadly. Yet greater integration of these spheres, such as in the form of un-earmarked funding and attention to underlying problems of chronic poverty, can promote more rapid recovery and reduce the gap in service provision between “emergency” and non/post-emergency situations, as in the response to the 2006 Indian Ocean tsunami [36]. Actors working on combined/simplified protocols should strive towards definitional clarity in terms of specific criteria for triggering these protocols, as well as articulate links to programs for managing childhood malnutrition in “non-emergency” or development contexts.

This study compared countries with different types of emergencies and at different stages of revising their national-level policies, providing insights into how diffusion of combined/simplified protocols varies in different contexts. Nonetheless this study had some limitations. Data were collected by a team of two researchers, one in Niger and northeast Nigeria, and the other in Somalia and South Sudan. To ensure congruity between the case studies, the two researchers held regular and frequent discussions, including debriefing during or shortly after field visits. Given that both researchers were employed by IRC (one as staff and one as a consultant), triangulation among data sources was used to mitigate any positive or negative respondent bias toward IRC. However, given IRC’s role in several Combined Protocol-related research studies and policy discussions, we cannot ensure all bias was removed. The lead author, staff of IRC, was a Principal Investigator of a prospective cohort study examining a version of the Combined Protocol in Mogadishu, Somalia, but is not actively involved in any national or global policy discussions on this topic. The senior author, a consultant, is not involved in any Combined Protocol-related activities beyond this study. Not all interviewees were able to be reached in all study countries due to the short duration of country visits and the frequent field travel of respondents. In Somalia in particular, security conditions made it difficult to reach many potential respondents, although additional data were collected with stakeholders based in Nairobi. Data collectors used additional phone interviews to reach saturation on almost all relevant points, however it remains possible that some perspectives were not fully represented. Finally, it can be difficult to elicit honest answers from respondents in policy studies, given that interviewees often see themselves as representing their institutions and may wish to advance certain policy or institutional positions. We addressed this limitation by training data collectors on appropriate interview techniques, triangulating between different respondents and data sources, and continuing interviews until we reached saturation on points of difference and/or contention.

## Conclusion

Countries experiencing conflict and other types of emergencies typically have complex governance environments, with varying degrees of central government control and a multiplicity of non-state actors collaborating in a humanitarian response. The large number of stakeholders involved in policy decision-making and service delivery creates a special imperative for transparent and clear communication from global policymakers about how the former can maximize the impact of their work. Ongoing work to provide coherent global guidance on combined/simplified protocols is therefore a welcome step to improve treatment and care for acutely malnourished children in emergency settings.

## Data Availability

The qualitative data from this study will not be made publicly available, due to the ease at which interviewees may be identified through the full transcripts, even if key sections are redacted.

## Abbreviations

ACF: Action Against Hunger
ALIMA: Alliance for International Medical Action
ECHO: European Civil Protection and Humanitarian Aid Operations
IRC: International Rescue Committee
NGO: Non-governmental organization
MAM: Moderate acute malnutrition
MOH: Ministry of Health
MUAC: Mid-upper arm circumference
OTP: Outpatient Treatment Programme
RUSF: Ready-to-use Supplementary Food
RUTF: Ready-to-Use Therapeutic Food
SAM: Severe acute malnutrition
TSFP: Targeted Supplementary Feeding Programme
WFP: World Food Programme
WHO: World Health Organization
U.N.: United Nations
